# Increasing Temperature and Microplastic Fibers Jointly Influence Soil Aggregation by Saprobic Fungi

**DOI:** 10.3389/fmicb.2019.02018

**Published:** 2019-09-06

**Authors:** Yun Liang, Anika Lehmann, Max-Bernhard Ballhausen, Ludo Muller, Matthias C. Rillig

**Affiliations:** ^1^Institut für Biologie, Freie Universität Berlin, Berlin, Germany; ^2^Berlin-Brandenburg Institute of Advanced Biodiversity Research, Berlin, Germany

**Keywords:** abrupt, gradual, temperature, microplastic, fungi, soil aggregation

## Abstract

Microplastic pollution and increasing temperature have potential to influence soil quality; yet little is known about their effects on soil aggregation, a key determinant of soil quality. Given the importance of fungi for soil aggregation, we investigated the impacts of increasing temperature and microplastic fibers on aggregation by carrying out a soil incubation experiment in which we inoculated soil individually with 5 specific strains of soil saprobic fungi. Our treatments were temperature (ambient temperature of 25°C or temperature increased by 3°C, abruptly versus gradually) and microplastic fibers (control and 0.4% w/w). We evaluated the percentage of water stable aggregates (WSA) and hydrolysis of fluorescein diacetate (FDA) as an indicator of fungal biomass. Microplastic fiber addition was the main factor influencing the WSA, decreasing the percentage of WSA except in soil incubated with strain RLCS 01, and mitigated the effects of temperature or even caused more pronounced decrease in WSA under increasing temperature. We also observed clear differences between temperature change patterns. Our study shows that the interactive effects of warming and microplastic fibers are important to consider when evaluating effects of global change on soil aggregation and potentially other soil processes.

## Introduction

Our soils are confronted with an unprecedented change: due to anthropogenic influence the climate is globally changing but also new threats of contamination are emerging (Allen et al., 2014; de Souza  Machado et al., 2018a). These changes act upon the soil system with far-reaching but poorly understood consequences on soil biota, soil functions [e.g., gas exchange, water infiltration, erosion resistance (Stewart and Hartge, 1995)] and ecosystem services [e.g., carbon storage, food production (Wheeler and von Braun, 2013; Crowther et al., 2016)]. A key aspect of soil to consider is its structure: the configuration and assemblage of soil particles, aggregates and associated pore networks (Lal, 1991). Soil structure can be affected by aspects of global change and in turn controls key aspects of soil biota and soil functions; thus, in order to mechanistically understand effects of global change on soil we need to assess consequences for soil structure and the process leading to soil structure, soil aggregation. The process of soil aggregation encompasses the process components formation, stabilization and disintegration; all working simultaneously under natural conditions giving rise to soil aggregates which represent building blocks of soil structure.

Soil aggregation is a biota-driven process with soil microbes in general and filamentous soil fungi in particular as substantial contributors (Forster, 1990; Degens, 1997; Lehmann and Rillig, 2015; Lehmann et al., 2017). Soil fungi contribute to and are affected by their complex and heterogeneous environment in multiple ways: during their foraging activities, hyphae of soil fungi entangle and enmesh soil particles (Tisdall, 1991; Daynes et al., 2012; Gupta and Germida, 2015) and aggregates, while also exuding exo-biopolymers functioning as binding agents (Chenu, 1989; Caesar-Tonthat, 2002; Daynes et al., 2012). Conversely, fungal growth and activity itself is modulated by the biotic, abiotic and spatial context of the soil matrix (Harris et al., 2003; Boswell et al., 2006).

It is widely acknowledged that temperature is a crucial factor determining activity of soil microbes (Pietikäinen et al., 2005), with higher temperature stimulating biological activities, such as respiration, growth rate, decomposition, extracellular enzyme activity, and secretion of metabolites (Zogg et al., 1997; Allison and Treseder, 2008; Dang et al., 2009; Bell et al., 2010); this also holds true for soil fungi (Jackson et al., 1991; Loera et al., 2011; A’Bear et al., 2012). Thereby, elevated temperature potentially influences soil processes driven by microbes, including soil aggregation. Nevertheless, data on the influence of soil warming on soil fungal contributions to soil aggregation are very limited. The limited data available suggest that with increasing soil temperature soil aggregate stability decreases (Rillig et al., 2002; Guan et al., 2018). The role of soil fungi in this decrease of soil aggregation with increasing temperature is not clear.

Studies on increasing temperature commonly increase temperature abruptly, which ignores the fact that temperature might also rise gradually in nature. The limited suite of studies applying such a gradual approach detected less pronounced effects in plant and soil microbe activity than under abrupt changes (for CO_2_ and salinity (Klironomos et al., 2005; Yan and Marschner, 2013). For soil microbes (e.g., arbuscular mycorrhiza fungi), shifts in community composition and functionality were detectable (Klironomos et al., 2005), suggesting that slower, more gradual rates of environmental change can result in more adapted and thus more resilient, final populations of organisms or fewer extinctions. In addition, a simulation study demonstrated specifically for soil warming that an abrupt change resulted in larger soil respiration than did gradual change (Shen et al., 2009). Considering these data, it is necessary to assess the effects of abrupt vs. gradual change when investigating the impact of increasing temperature on fungal contributions to soil aggregation, because such a comparison may yield more robust insights.

Soils are exposed to a multitude of global change factors some of which recently moved into research focus; among these is microplastics - a group of pervasive, ubiquitous, anthropogenic contaminants (de Souza  Machado et al., 2018a). Microplastics comprise chemically diverse polymers of varying shapes and structures, with a size range from 5 mm to 1 μm which are produced as (primary) or fragmented into (secondary) micro-sized plastic particles via environmental factors (Hartmann et al., 2019). Microplastics can be found world-wide not only in marine but also terrestrial ecosystems, which received considerable attention recently (Rillig, 2012; Huerta Lwanga et al., 2016; Bläsing and Amelung, 2018). The limited data available so far suggests that microplastic particles, especially fibers, have indirect effects on soil aggregation via the soil microbial pathway by physically changing soil properties [e.g., decreasing soil bulk density (de Souza  Machado et al., 2018b)]. By changing the conditions in the soil matrix, a shift in fungal growth and activity can be expected. There are so far no studies evaluating the effect of microplastics on soil fungi; a significant gap in our knowledge, which has to be approached to understand microplastic impact on soil aggregation. We assume that lower bulk density, particularly due to microplastic fiber addition, leads to higher aeration-dependent microbial activities, moreover, the increased pore space likely provides favorable conditions for hyphal extension (Elliott et al., 1988; Wang et al., 2017).

Although environmental factors affect soils in combination, studies tend to test these factors in isolation. This hampers our understanding of potential interactive effects on the targeted study systems. To contribute to the identified research gaps in effects of environmental factors on fungal mediated soil aggregation, we conducted a laboratory study with soil inoculated with filamentous soil fungal strains and microplastic fiber and temperature (with both abrupt and gradual increase) treatments. We aimed to investigate interactive effects of two global change drivers of significant importance for soil and fungal systems. We test the following hypotheses: (1) Rising temperature will lead to higher fungal activity resulting in higher fungal contribution to soil aggregation as compared to control settings. (2) Gradually rising temperatures will affect fungal activity and soil aggregation less than abrupt temperature change. (3) Microplastic fibers will promote fungal activity and fungal contributions to soil aggregation. (4) Microplastic fibers and increasing temperature will interact in their effects on fungal activity and soil aggregation.

## Materials and Methods

### Microfiber

In this experiment, we focus on microplastic fibers, since in a previous experiment plastic fibers elicited stronger effects on soil aggregation than fragments or beads (de Souza  Machado et al., 2018b). Additionally, recent studies found that atmospheric deposition of microplastic fibers is an important source of soil contamination (Dris et al., 2016; Zhang and Liu, 2018; Henry et al., 2019). From the many available polymer types, we chose polyacrylic (PAN) fibers, which are produced from acrylonitrile. Polyacrylic fibers are easy to process and can affect soil aggregation (de Souza  Machado et al., 2018b). We produced microfibers by manually cutting 100% acrylic “Bravo” yarn (schachenmayr.com) into length ranges from 0.37 to 3.14 mm (Supplementary Figure S1). The diameter of these polyacrylic fibers was 0.026 ± 0.005 mm. The microfibers were sterilized by microwaving and subsequently added to the soil as 0.4% (w/w), 0.4% was determined as the upper limit concentration in previous study in our lab, which was determined based on the highest concentration at which soils experienced minor changes in volume after the addition of linear microplastics (de Souza  Machado et al., 2018b). The sterilized fibers were placed on PDA (see below) plates, no contamination was observed after 7 days.

### Fungi Inoculum Preparation

We selected five fungal strains from a set of filamentous fungi maintained in our lab (Andrade-Linares et al., 2016), the method of identification is given in Supplementary Table S1, originally isolated from a natural semi-arid grassland (Mallnow, Lebus, Brandenburg, Germany, 52°27.7780′ N, 14°29.3490′ E): RLCS 01, RLCS 05, RLCS 06, RLCS 07, RLCS 08, the species are: *Mucor fragilis, Fusarium sp., Chaetomium angustispirale, Amphisphaeriaceae strain 1, Gibberella tricincta*, respectively (Supplementary Table S1). Instead of referring to a species name, we address our strains solely with the identifier RLCS following with the strain-specific number. Strains are sorted by colony radial extension rate from high to low (Supplementary Table S1). The fungal strain information table is in Supplementary Table S1. The fungal strains were filamentous, saprobic fungi, selected for comparable growth rate on potato dextrose agar (PDA X931.2, Roth, Germany) (Zheng et al., 2018), and optimum growth temperature around 25°C (data not shown here). These isolates had different ability to form soil aggregates (Lehmann et al., 2019b), RLCS 01 is the poorest soil aggregator while RLCS 08 is the best. In choosing these isolates, we minimize confounding effects by differences in produced fungal biomass, while covering variance of soil aggregation capability. Fungi were cultured in potato dextrose broth (PDB CP74.2, Roth, Germany) for 4 days on a rotary shaker (New Brunswick^TM^, Eppendorf) at 150 rpm. Hyphal fragments for inoculation were produced by disrupting the mycelium with glass beads (diameter = 0.25–0.5 mm; Roth, Germany) shaken on a vortex mixer at highest speed for 1 min (Vortex-Genie, Scientific Industries, United States). The resulting suspension was passed through a 20 μm nylon membrane to retrieve fungal fragments of a homogeneous size. In order to minimize the difference in propagule numbers, fungal mycelium fragments were diluted in PDB to a concentration of 40–100 fragments per 20 μl. In a preliminary test, we plated the mycelium fragment solutions of the different strains and counted the emerging colonies (Supplementary Table S2). For each strain, we prepared final inoculum suspensions with the appropriate dilution factor. We used 20 μl of mycelium fragment-PDB suspension for inoculation and 20 μL of mycelium-free PDB for the controls.

### Incubated Soil

Fresh soil was collected from Mallnow, Lebus, a dry grassland in a natural reserve (Brandenburg, Germany, 52°27.7780′ N, 14°29.3490′ E) characterized as a sandy loam soil texture (Horn et al., 2015), from which the focal fungal strains had been originally isolated In this study, soil was sieved (1 mm) and thoroughly homogenized; this method is commonly used to measure macroaggregate formation in laboratory incubations (De Gryze et al., 2006). We placed 10 g (±0.01 g) of the soil in test tubes which were autoclaved twice (121°C for 20 min). After drying the soil at 60°C, we transferred the sterilized soil into Petri dishes (60 × 15 mm) to mix the sterilized soil with 40 mg of microfiber and 20 μl of fungal homogenate, depending on the treatment. The soil mixture was uniformly wetted with sterilized distilled water amended with glucose to keep water content at 80% water holding capacity and to ensure that every microcosm received 1.89 mg C-glucose to stimulate fungal growth. Glucose as an easily decomposable substrate that can rapidly stimulate the growth of soil microorganisms and has no direct effect on macroaggregation (Abiven et al., 2008), and our own preliminary data (not shown here) also confirmed this. Controls without inoculation also received the same amount of water and glucose.

All samples were sealed with parafilm and placed into plastic boxes with covers. We placed wet paper towels inside the boxes to maintain high air humidity to prevent the soil from drying. Samples were incubated for 42 days during which the temperature treatments were applied.

### Temperature Treatment

We incubated all microcosms at ambient temperature (25°C) for the first 7 days to let inoculants establish. To realize the gradually rising temperature, we increased temperature by 3°C from 25°C to 28°C at the speed of 0.15°C day^–1^ from day 8 to day 27. For the abrupt temperature treatment, the 3°C increase was applied on day 18. This difference in timing ensured that the mean temperature was the same for the gradual and abrupt temperature treatment (Supplementary Figure S2). We increased temperature by 3°C based on climate models, which predict that the mean annual global surface temperature will increase by 1–3.5°C until 2100 (Beckage et al., 2018).

### Experimental Design

Each of the five fungal isolates and the control were exposed to the combinations of microfiber [yes (M)/no (C)] and temperature (ambient (0), abrupt (+ 3abrupt) and gradual (+ 3gradual)), resulting in 6 treatments: T_0_-C, T_0_-M, T_+__3__*abrupt*_-C, T_+__3__*abrupt*_-M, T_+__3__*gradual*_-C, T_+__3__*gradual*_-M. Each treatment had 7 replicates, for a total of 252 experimental units. The units under elevated temperature were placed in three independent incubators split in groups of three and two-times two units. By this approach, we were able to also replicate increased temperature and account for the variability among incubators.

### Aggregate Stability

We dried soil samples at 40°C and then sieved them (2 mm). Before wet-sieving, 4.0 g dry soil was placed into sieves for capillary rewetting and subsequently submerged in deionized water for 5 min. We used 0.25 mm sieves to test the stability of the soil fraction >0.25 mm (macroaggregate) against water as disintegrating force. For the test, sieves carrying the wetted soil samples were placed in a wet-sieving machine (Eijkelkamp, Netherlands) for 3 min. The fractions left on the sieves were dried at 60°C for 24 h. The coarse matter (sand and organic matter fraction) was extracted before calculation of the percent water stable aggregates (WSA):

$$\%\mathrm{WSA}=\frac{\mathrm{water}\,\mathrm{stable}\,\mathrm{fraction}-\mathrm{coarse}\,\mathrm{matter}}{4\,g-\mathrm{coarse}\,\mathrm{matter}}$$

### Hydrolysis of Fluorescein Diacetate

We measured he fluorescein diacetate (FDA) hydrolytic activity to indicate fungal activity, which is considered as an indicator of fungal biomass (Gaspar et al., 2001). We quantified the hydrolysis of fluorescein diacetate (FDA, Sigma-Aldrich) by adding 0.75 ml of 100 mM of potassium phosphate buffer (pH 7.6) and 0.1 ml of 2 mg/ml FDA (Adam and Duncan, 2001) to 0.5 g of dry soil. The reaction mixture was placed on a shaker (New Brunswick^TM^, Eppendorf) at 150 rpm at 30°C for 2 h. The reaction was terminated by adding 0.75 ml of acetone (Roth, Germany). After shaking and centrifugation of samples for 5 min at 3000 rpm, the extracted fluorescein was determined at 490 nm by spectrophotometry (UV-3100 PC, VWRTM, Germany).

### Statistical Analysis

We analyzed the effects of elevated temperature, microfiber addition and fungal species by using three-way ANOVAs. We used Shapiro–Wilk test and Bartlett test to check the normality of residuals and the homogeneity of variances, respectively, with a *p*-value cutoff of 0.05. We compared the difference between the treatments according to Duncan’s test or Student’s *t*-test at a probability level of 5%. All statistics were conducted in R (R Core Team, 2017) with the basic packages, while the plots were created with the graphic package “ggplot2” (Hadley and Winston, 2016).

## Results

### Fungal Contributions to Soil Aggregation

**FIGURE 1:**
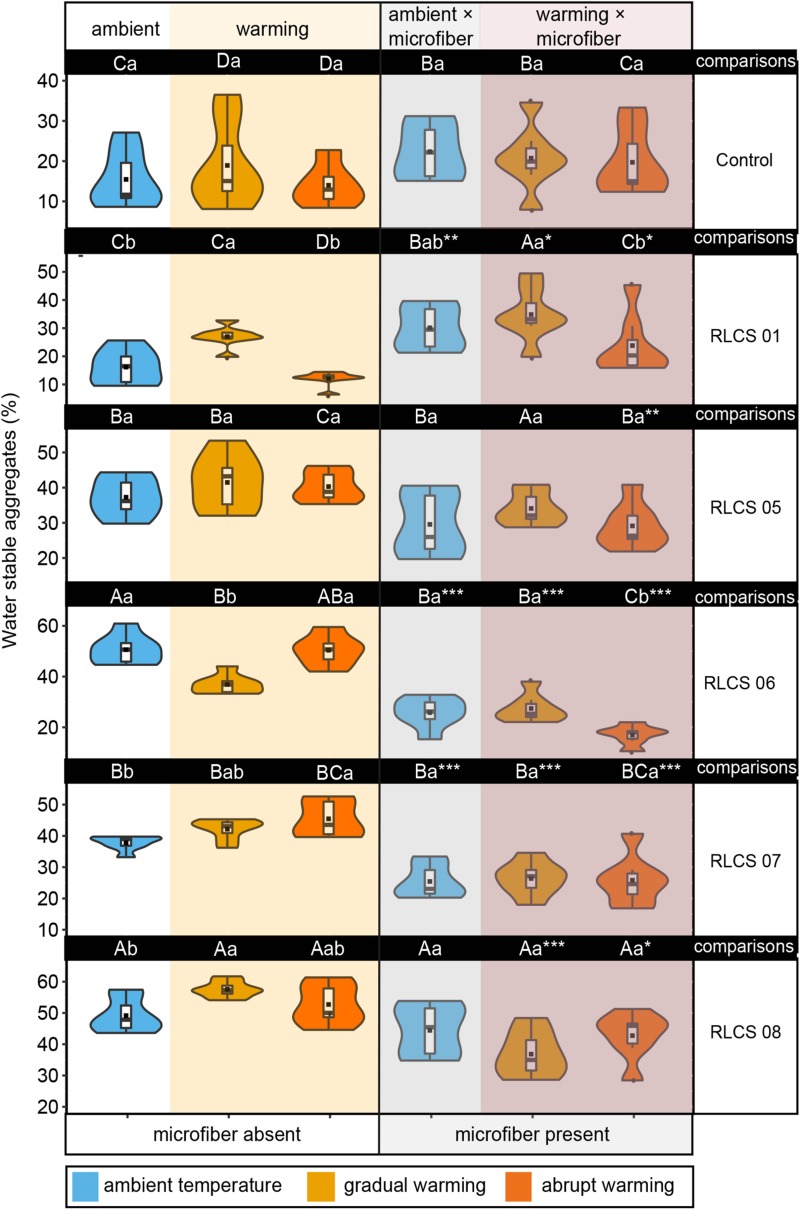
The effects of temperature and microplastic fiber addition on the percentage of water stable aggregates depicted as violin/box plots. Different capital letters indicate significant differences (Duncan’s test, *p* < 0.05) among different fungi for the same treatment (i.e., in columns), and lowercase letters indicate significant differences (Duncan’s test, *p* < 0.05) among temperature treatments in terms of microplastic being present or absent (that is, referring to each part of a row). Significant differences (*t*-test, ^∗^*p* < 0.05, ^∗∗^*p* < 0.01, and ^∗∗∗^*p* < 0.001, respectively) between microplastic being present and absent within the same temperature treatment. The dots (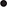) represent outliers, squares (■) represent means.

We investigated under ambient, non-contaminated conditions how fungal inoculation affected soil aggregation. We detected a significantly higher (*p* < 0.05) macroaggregate stability than in control samples with the exception of the strain RLCS 01 (Figure 1 and Supplementary Table S3), the strain RLCS 05 had the strongest positive effect (Supplementary Table S3) leading to a 227.49% increase in the percentage of WSA. The ability to form stable aggregates at 25°C varies among fungi.

### Temperature Effects on Fungal Contributions to Soil Aggregation

Elevated temperature alone caused increase in WSA for soil with RLCS 01, RLCS 07, and RLCS 08, caused no change in WSA for soil with RLCS 05. Nevertheless, WSA of soil with RLCS 06 significantly decreased (*p* < 0.05) under gradually elevated temperature.

In case of soil with RLCS 01 and RLCS 06, we observed a significant difference (*p* < 0.05) in the percentage of WSA between abrupt change and gradual change.

### Plastic Microfiber Effects on Fungal Contribution to Soil Aggregation

Regardless of rising temperature, microfiber addition dramatically decreased the percentage of WSA in soil with RLCS 06 and RLCS 07 by 49.08 and 32.69%, respectively, but significantly increased the percentage of WSA in soil with RLCS 01 by 86.33%. Microfiber addition alone, in the absence of fungi, had no significant effect (*p* > 0.05) on the percentage of WSA.

### Interactive Effects of Temperature and Plastic Microfibers on Fungal Contributions to Soil Aggregation

We observed significant interactive effects between elevated temperature and plastic microfibers (Table 1). For soil with RLCS 01, RLCS 07 and RLCS 08, rising temperature caused a significant increase (*p* < 0.05) in the percentage of WSA for treatment T_+__3__*abrupt*_-C, T_+__3__*gradual*_-C, T_+__3__*gradual*_-C, compared to control (T_0_-C). Such increases disappeared in the presence of microfibers, resulting in no significant difference in WSA between ambient and elevated temperature.

**TABLE 1:** ANOVA results for the effects of fungi, temperature, plastic microfibers and the interaction of these factors on percentage of water-stable macroaggregates.

**Source**	***F* value**	***P* value**
Fungi	96.489	0.0001^∗∗∗^
Temperature	4.066	0.018^∗^
Microfibers	68.136	0.0001^∗∗∗^
Fungi × Temperature	3.322	0.0005^∗∗∗^
Fungi × Microfibers	38.384	0.0001^∗∗∗^
Temperature × Microfibers	2.351	0.098
Fungi × Temperature × Microfibers	3.438	0.0003^∗∗∗^

*Significant differences ^∗^*p* < 0.05, ^∗∗^*p* < 0.01, and ^∗∗∗^*p* < 0.001, respectively.*

The effects of rising temperature on WSA might even shift in the presence of microfibers. For soil with RLCS 07 and RLCS 08, abruptly and gradually elevated temperature, which respectively, caused the highest stability, unexpectedly led to the lowest WSA in the presence of microfibers, indicating that effects of rising temperature might even turn to negative, leading to greater loss in the percentage of WSA than microfiber alone. Moreover, for soil with RLCS 06, though gradually elevated temperature had negative effect, no significant difference (*p* < 0.05) was observed in the percentage of WSA between T_+__3__*agradual*_-M and T_0_-M in the presence of microfibers.

In our study, the effect of microfibers also depended on temperature change patterns. Under abruptly elevated temperature, for soil with RLCS 06, microfibers caused substantial decreases in the percentage of WSA by 66.3%, which were higher than the decreases of 25.68% under gradually elevated temperature. Under abruptly elevated temperature, for soil with RLCS 08, microfibers caused a greater decrease in the percentage of WSA than under abruptly increased temperature.

### FDA Hydrolysis Activity

**FIGURE 2:**
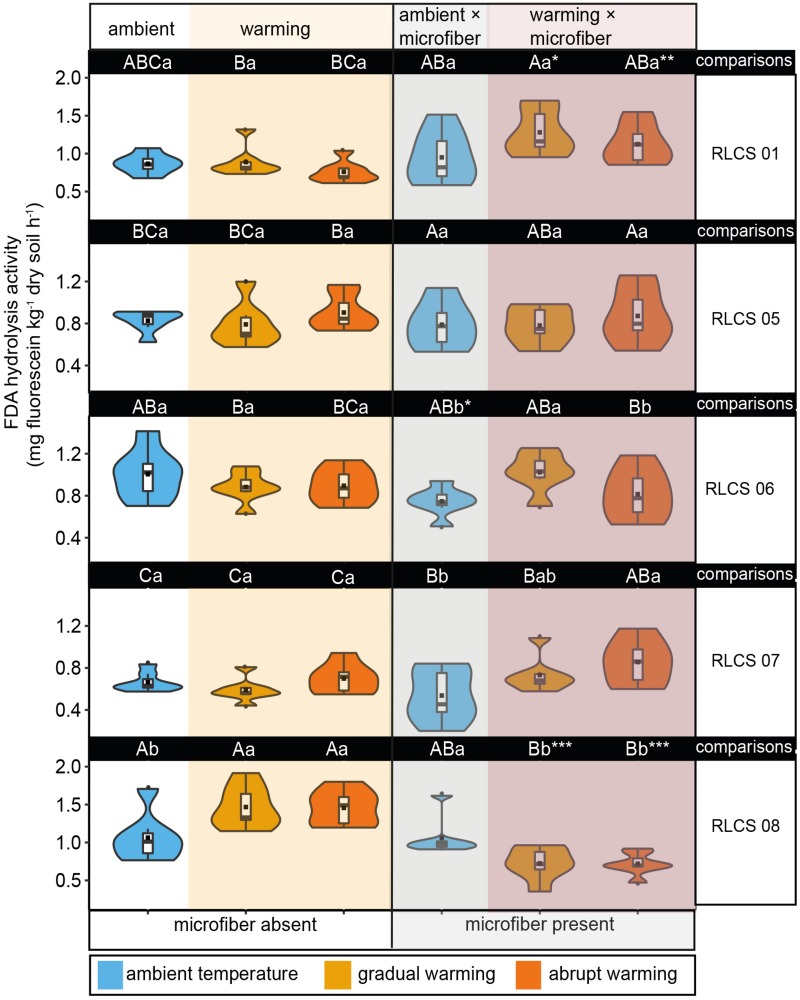
The effects of temperature and microplastic fiber addition on FDA hydrolysis activity depicted as violin/box plots. Different capital letters indicate significant differences (Duncan’s test, *p* < 0.05) among different fungi for the same treatment (i.e., in columns), and lowercase letters indicate significant differences (Duncan’s test, *p* < 0.05) among temperature treatments in terms of microplastic being present or absent (that is, referring to each part of a row). Significant differences (*t*-test, *p* < 0.05, ^∗∗^*p* < 0.01, and ^∗∗∗^*p* < 0.001, respectively) between microplastic being present and absent within the same temperature treatment. The dots (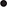) represent outliers, squares (■) represent means.

The interactive effect of increasing temperature and microfibers on FDA hydrolysis activity was significant (Table 2). Generally, FDA hydrolysis activity was positively correlated with WSA, especially for soil with RLCS 01 (*r* = 0.44, *p* = 0.0039) and RLCS 08 (*r* = 0.61, *p* = 2.1e-05). Rising temperature alone caused significant increase (*p* < 0.05) in FDA hydrolysis activity for soil with RLCS 01, RLCS 07 and RLCS 08, which is consistent with the WSA data. Microfiber alone led to higher FDA in soil with RLCS 01 while causing lower FDA in soil with RLCS 06 and RLCS 08 (Figure 2).

**TABLE 2:** ANOVA results for the effects of fungi, temperature (abrupt and gradually rising temperatures), plastic microfiber and the interaction of these factors on FDA hydrolysis activity of soil.

**Source**	***F* value**	**Pr (>*F*)**
Fungi	22.58	0.0001^∗∗∗^
Temperature	3.12	0.047^∗^
Microfibers	2.40	0.12
Fungi × Temperature	1.44	0.18
Fungi × Microfibers	18.94	0.0001^∗∗∗^
Temperature × Microfibers	0.34	0.71
Fungi × Temperature × Microfibers	7.21	0.0001^∗∗∗^

*Significant differences ^∗^*p* < 0.05,^∗∗^*p* < 0.01 and ^∗∗∗^*p* < 0.001, respectively.*

## Discussion

We here present data from a laboratory study investigating for the first time the impact of plastic microfibers and temperature increase on fungal effects on soil aggregation. We find that effects were dependent on the fungal species, and that microfibers and temperature interacted in complex ways. In the following, we discuss results in a progression from main effects to their interaction.

We found the ability to form stable aggregates at ambient temperature varies among fungi. Previous work demonstrated that traits of fungi have different roles in formation, stabilization and disintegration of aggregation (Lehmann and Rillig, 2015), and thus fungi that vary in traits are expected to differently affect WSA. Basically, fungal strains of the Ascomycota could form aggregates efficiently while strains belonged to the Mucoromycota are poor aggregate formers (Lynch and Elliott, 1983; Tisdall et al., 2012). According to our previous study, the traits of fungi contributing to aggregate formation include high biomass density, large hyphal diameters, and low leucine aminopeptidase activity (Lehmann et al., 2019b).

The increase in WSA due to increasing temperature might be attributed to enhanced hyphal growth and higher levels of secretion of bindings agents, however, warming also accelerates decomposition of such binding agents, which are important for forming stable soil aggregates (Tisdall and Oades, 1982), resulting in breakdown of aggregates. Given these different mechanisms, the overall effects might be species-specific. RLCS 06 (*Chaetomium angustispirale*) as a cellulolytic fungus has a strong ability to degrade cellulose (Sahab Yadav and Bagool, 2015), and thus it is possible that RLCS 06 accelerated decomposition of soil organic matter under gradually increased temperature, resulting in less WSA.

Even though we hypothesized that gradually increased temperature would lead to milder effects on WSA, changes in the percentage of WSA caused by gradually rising temperature were still substantial in some cases, which probably is due to the rate of change being relatively high (0.15°C day^–1^), thus potentially not permitting fungi to adjust to the warming. However, the significant difference (*p* < 0.05) in the percentage of WSA between temperature change patterns, for soil with RLCS 01 and RLCS 06, still underscored that gradually rising temperature should be explored more in laboratory warming experiments to assess differential effects.

Decreases in WSA due to microfiber addition were observed in previous studies (de Souza  Machado et al., 2018b; Zhang and Liu, 2018). Polyacrylic fibers significantly decreased the amount of water stable aggregates (de Souza  Machado et al., 2018b). Possible reasons include plastic fibers preventing microaggregates from effectively being integrated into macroaggregates (Zhang and Liu, 2018), and the inclusion of microfibers within macroaggregates (Bläsing and Amelung, 2018), finally leading to less stable macroaggregates. However, RLCS 01 led to higher WSA when microfibers were added, a response for which an explanation is not clear. Microfiber increased FDA in soil with RLCS 01, therefore this strain was likely able to maintain or even have higher activity in the presence of microfiber, which may partially explain the observed effect. Previous studies found some fungal species could degrade plastic (Cappitelli and Sorlini, 2008; Moharir and Kumar, 2019)The polyacrylic polymers used here were found to be mineralized by white- rot fungi (Sutherland et al., 1997) which are capable of degrading the most recalcitrant biological polymers (i.e., lignin) (Kirk et al., 1978). Nevertheless, for our experimental setup, we do not use white- rot fungi, and we also do not see a confounding effect by the potential carbon utilization from polyacrylic fibers by our fungi. We provided sufficient, easily available organic C for the fungi here, such that any utilization of C from the fibers should have played a minor role.

Microfiber addition alone, in the absence of fungi, had no significant effect (*p* > 0.05) on the percentage of WSA. This suggests that effects of microfibers on soil aggregation require the presence of soil biota (Lehmann et al., 2019a); the latter are necessary to build soil aggregates, in our experiment fungi, but incubating soil with just the microfibers evidently had no deleterious direct (physical) effects on WSA.

Microfiber addition negated the positive effect of rising temperature on WSA in the case RLCS 01, RLCS 07 and RLCS 08. Nonetheless, the effects of these two factors were not additive. Regarding RLCS 07 and RLCS 08, effects of rising temperature even turned out to be negative in the presence of microfibers, leading to greater loss in the percentage of WSA than microfiber alone. We currently do not know what caused this effect. This was most likely because rising temperature led to faster fungal growth together with faster decomposition of soil organic matter increasing the production of binding agents for soil aggregates, thus resulting in more aggregate disintegration (Rillig et al., 2002) and formation under rising temperature, in other words a greater turnover of macroaggregates. Microfibers can be entrapped within macroaggregates (Bläsing and Amelung, 2018), perhaps leading to less stable macroaggregates. Thereby, when higher temperatures benefited WSA, microfiber fibers might be incorporated into newly forming macroaggregates, resulting in less stable aggregates. Moreover, for soil with RLCS 06, the negative effects of rising temperature disappeared in the presence of microfibers, it is possible that RLCS 06 accelerated decomposition of soil organic matter under gradually elevated temperature, thus fewer microfibers became incorporated into aggregates when the effect of gradually elevated temperature on WSA was negative; this could explain why the negative effect of gradually elevated temperature on WSA disappeared in the presence of microfiber.

In our study, the effect of microfibers also depended on temperature change patterns. We hypothesized that the effect of microfibers on WSA depended on how WSA responded to the temperature increase pattern.

An increase in FDA hydrolysis activity caused by rising temperature was shown previously (Sinsabaugh et al., 2008; Baldrian et al., 2013), indicating that warming increased fungal activity. Nevertheless, the differences of FDA between rising temperature and ambient temperature are not statistically clear except for soil with RLCS 08. We suggest that even small changes in FDA might cause large changes in the percentage of WSA, or that fungal effects not captured by FDA are important. The rising temperature might enhance the expression of traits that benefit formation and stabilization of aggregates by certain fungi: RLCS 01, RLCS 07, and RLCS 08. Such traits include the stability and longevity of hyphae, entanglement potential of soil particles (Lehmann and Rillig, 2015; Rillig et al., 2015) and also higher secretion rate of protein and metabolic products in warmer conditions (Papagianni, 2004).

Further research is needed to measure fungi-caused decomposition and fungal biomass density, in order to decouple the negative effect and positive effect of fungi on soil aggregation formation. Therefore, it would be desirable to develop a method which can distinguish plastic C from soil C, such as using ^13^C- labeled microplastic (Zumstein et al., 2018).

## Conclusion

In our study, plastic microfibers might eliminate the positive effect of temperature on soil aggregation, and could even lead to greater losses in the percentage of WSA. Thus, we emphasize the importance of considering the potentially strong decrease due to interactive effects between microplastics and global warming. Our study lends further support to general findings of prior research on the interactive effects of environmental factors on soil function (García et al., 2018; Tekin et al., 2018), suggesting strongly that global change effects should be analyzed not only as single factors but also in combination.

We found that sensitivities to the environmental factors differed among fungal species, and thus our study opens the door to the examination of the behavior of fungal communities when exposed to this combination of environmental factors.

## Data Availability

The raw data supporting the conclusions of this manuscript will be made available by the authors, without undue reservation, to any qualified researcher.

## Author Contributions

YL designed and performed the study, conceived and performed the data analyses. YL, AL, M-BB, and MR wrote the manuscript. All authors contributed to the final version of the manuscript.

## Conflict of Interest Statement

The authors declare that the research was conducted in the absence of any commercial or financial relationships that could be construed as a potential conflict of interest.
