# Supplementary material for: Increasing Temperature and Microplastic Fibers Jointly Influence Soil Aggregation by Saprobic Fungi
Source: Front Microbiol. 2019 Sep 6;10:2018. doi: 10.3389/fmicb.2019.02018 (PMC6742716; doi:10.3389/fmicb.2019.02018)
Supplement: Supplementary file 1 [file Data_Sheet_1.docx]

Supplementary Material

## Supplementary Figures
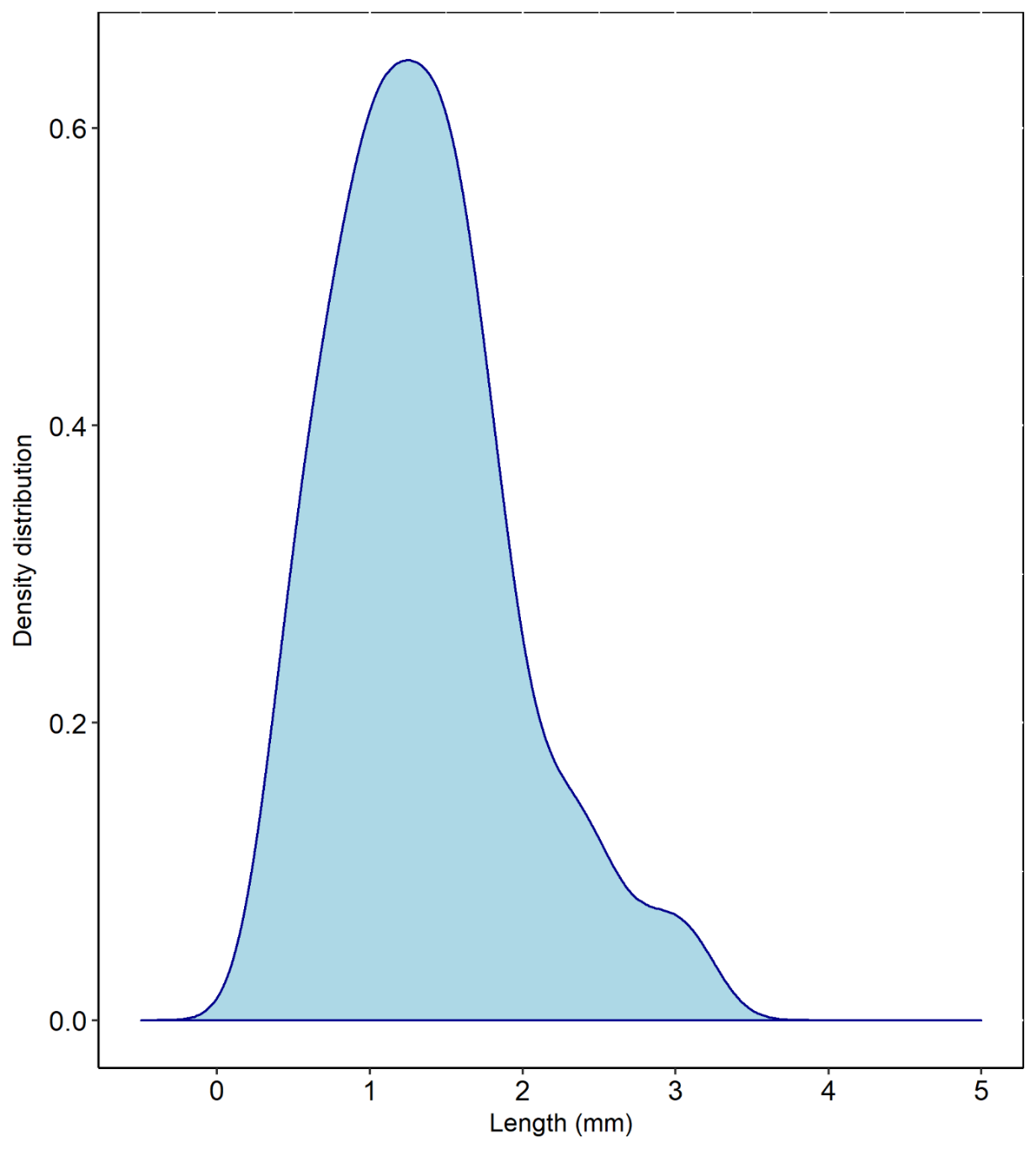
Supplementary Figure 1. Microfiber size distribution. Polyacrylic fibers (N= 113) were measured under a microscope.


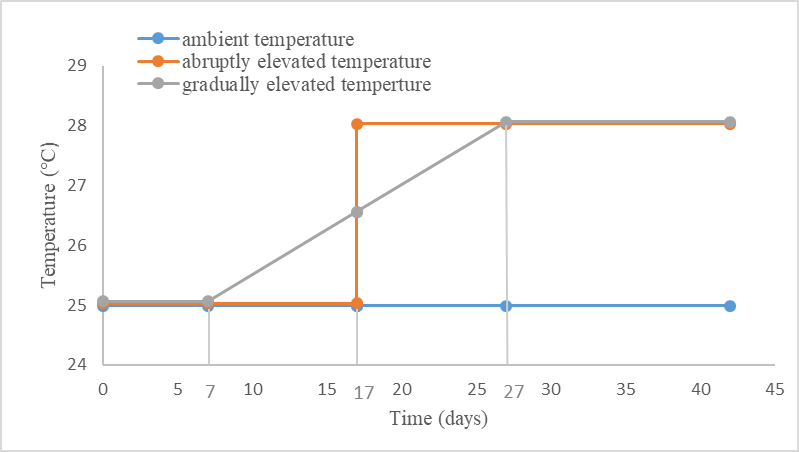


**Supplementary Figure 2.** Depiction of temperature increase patterns. The ambient temperature was set at 25℃ while the elevated temperature was targeted at 28℃. For temperature elevation, two distinct increase patterns (abrupt and gradual) were explored. The two temperature elevation patterns started on different days (abrupt pattern: start on day 18; gradual pattern: temperature was increased by 0.15℃/day from day 8 to day 27), but both lasted until day 42 Following these two patterns, the average temperatures of abrupt and gradual increase are the same.

## Supplementary Table

**Supplementary Table 1.** Information about phylum, order and Genbank and Deutsche Sammlung von Mikroorganismen und Zellkulturen (German Collection of Microorganisms and Cell Cultures GmbH, DSMZ) accession numbers of the five fungal strains used in this study.

| **Fungal strain**  **ID** | **Taxonomic identification** | **Family** | **Order** | **Phylum** | **DSMZ** | **Radial colony growth rate (µm/h)** |
| --- | --- | --- | --- | --- | --- | --- |
| RLCS 01 | *Mucor fragilis* | Mucoraceae | Mucorales | Mucoromycotina | DSM 100293 | 372.7625 |
| RLCS 05 | *Fusarium sp.* | Nectriaceae | Hypocreales | Ascomycota | DSM 100403 | 215.1519 |
| RLCS 06 | *Chaetomium angustispirale* | Chaetomiaceae | Sordariales | Ascomycota | DSM 100400 | 198.463 |
| RLCS 07 | *Amphisphaeriaceae strain 1* | Amphisphaeriaceae | Xylariales | Ascomycota | DSM 100284 | 196.82174 |
| RLCS 08 | *Gibberella tricincta* | Nectriaceae | Hypocreales | Ascomycota | DSM 100325 | 183.00533 |

The identification of fungal isolates was done based on the complete intergenic transcribed spacer (ITS) and a part of the large rRNA subunit (LSU). Each region was aligned independently, using AlignSeqs in the R package DECIPHER 2.0. Aligned subregion sequences were concatenated. Pairwise distances from sequences were calculated using the JC69 evolutionary model and used to construct a neighbor-joining tree, with the dist.ml and NJ functions respectively, of the R package “phangorn” 2.5.5. Root was placed at the midpoint of the longest path between any two tips. Taxonomic annotations of the fungal isolates were based on each subregion. We used the Naive Bayesian Classifier as implemented in the R package “dada2” against UNITE database for ITS1 or ITS2, and against the RDP LSU database for LSU. A confidence threshold was calculated with bootstrap analysis and an annotation was deemed valid when supported in 80% of the bootstraps. The best resolved taxonomic annotation among the regions was chosen. In the case of a conflict of taxonomic annotations between regions, priority was given to ITS1 or ITS2 because UNITE is more complete than the RDP LSU database.

**Supplementary Table 2.** Plate counting of homogenates (replicates= 3, per fungal strain).

| **Fungal strain** | **Original numbers of fungal fragments** | **Numbers of fungal fragments after dilution** |
| --- | --- | --- |
| RLCS 01 | ca.100 | ca.100 |
| RLCS 05 | 400, 500, 400 | ca.40 |
| RLCS 06 | 35, 44, 46 | No dilution |
| RLCS 07 | 72,71,70 | ca.70 |
| RLCS 08 | 150, 145, 200 | ca.75 |

**Supplementary Table 3.** Percentage of water stable macroaggregates among different treatments.

| **Fungal species** | **Microfiber absent** | | |  | **Microfiber present** | | |
| --- | --- | --- | --- | --- | --- | --- | --- |
|  | Ambient temperature | Gradually elevated  temperature | Abruptly elevated temperature |  | Ambient temperature | Gradually elevated  temperature | Abruptly elevated temperature |
| Control | 15.46±6.90  Ca | 18.93±10.49  Da | 14.00±5.20  Da |  | 22.36±6.87  Ba | 20.55±8.09  Ba | 19.71±8.14  Ca |
| RLCS 01 | 16.17±6.12  Cb | 27.00±3.85  Ca | 12.03±2.61  Db |  | 30.13±7.74  Bab | 34.84±9.42  Aa | 23.81±10.74  Cb |
| RLCS 05 | 37.27±5.46  Ba | 41.48±7.72  Ba | 40.30±4.23  Ca |  | 29.55±8.88  Ba | 34.09±4.70  Aa | 29.13±6.36  Ba |
| RLCS 06 | 50.63±6.36  Aa | 36.91±3.78  Bb | 50.38±6.11  ABa |  | 25.78±5.89  Ba | 27.43±5.50  Ba | 16.98±3.90  Cb |
| RLCS 07 | 37.78±2.35  Bb | 42.16±3.30  Bab | 45.46±5.99  BCa |  | 25.43±5.27  Ba | 26.38±5.36  Ba | 25.82±7.88  BCa |
| RLCS 08 | 49.15±5.55  Ab | 57.55±2.59  Aa | 52.71±6.28  Aab |  | 44.43±8.17  Aa | 36.84±7.24  Aa | 42.75±7.46  Aa |

Data are presented as mean and standard deviation. Different uppercase letters indicate significant differences (Duncan’s test) among different fungi (p < 0.05). Different lowercase letters indicate significant differences (Duncan’s test) among temperature treatments in terms of microfiber present or absent (P< 0.05).
